# The Lights and Shadows of Medical Life

**Published:** 1853-05

**Authors:** 


*The Lights and Shadows of Medical Life.*—We fully concur with our correspondent below, as respects the propriety of contemplating the joys, as well as sorrows pertaining to the life of the medical practitioner. And, in our opinion, it is the fault of the physician himself if the former do not preponderate. The grateful appreciation of professional services, the social relations founded thereon, and the inward satisfaction accompanying the successful management of diseases, more than overbalance the peculiar annoyances incident to medical life, not a few of which proceed from the jealousies and malignity of the small proportion of unworthy members who disgrace the medical profession:

*April* 19*th*, 1853.

Mr. Editor,—Perhaps you have read some of the "Sketches from the every-day experience of a Young Doctor." I have, and also the life-like narrative of Dr. G. Saultz; and both brought reminiscences of so gloomy a character, that I was glad to turn to the other side of the picture where Doctors are in luck, to prevent a fit of the blues. Do not the following, which have come to my lot, furnish a spice to the life of a physician so agreeable as to remove that disgust which frequent abuse often develops.

Had you not better mix in something of the kind to keep the readers of the Journal from pouting.

Dr. ———,—Please to accept the accompanying trifle, (a valuable diamond brooch,) as a token of that gratitude which your kindness and fidelity in my recent sickness, has inspired.

This pin was long worn by a most valued relative, and since his decease I have found no friend whom I would so well like to see wear it.

Respectfully yours, &c., M. W.

Dr. ———,—Dear Sir: Please accept this book, (a ten dollar Bible,) as a token of friendship from one who feels herself very much indebted for your kind services while in affliction. L. H.

BIEN HENREUX, M. D.

